# Regulation of CaMKII signaling in cardiovascular disease

**DOI:** 10.3389/fphar.2015.00178

**Published:** 2015-08-25

**Authors:** Mariya Y. Mollova, Hugo A. Katus, Johannes Backs

**Affiliations:** ^1^Research Unit Cardiac Epigenetics, Department of Cardiology, Angiology and Pneumology, University of Heidelberg, Heidelberg, Germany; ^2^Department of Cardiology, Angiology and Pneumology, University of Heidelberg, Heidelberg, Germany; ^3^Partner Site Heidelberg/Mannheim, German Center for Cardiovascular Research, Heidelberg, Germany

**Keywords:** CaMKII, heart failure, post-translational modifications, enzymatic activity, sub-cellular localization

## Abstract

Heart failure (HF) is a major cause of death in the developed countries (Murray and Lopez, 1996; Koitabashi and Kass, 2012). Adverse cardiac remodeling that precedes heart muscle dysfunction is characterized by a myriad of molecular changes affecting the cardiomyocyte. Among these, alterations in protein kinase pathways play often an important mediator role since they link upstream pathologic stress signaling with downstream regulatory programs and thus affect both the structural and functional integrity of the heart muscle. In the context of cardiac disease, a profound understanding for the overriding mechanisms that regulate protein kinase activity (protein-protein interactions, post-translational modifications, or targeting via anchoring proteins) is crucial for the development of specific and effective pharmacological treatment strategies targeting the failing myocardium. In this review, we focus on several mechanisms of upstream regulation of Ca^2+^-calmodulin-dependent protein kinase II that play a relevant pathophysiological role in the development and progression of cardiovascular disease; precise targeting of these mechanisms might therefore represent novel and promising tools for prevention and treatment of HF.

## Introduction

The human genome consists of approximately 500 different protein kinase genes that ultimately modify an estimated 30% of the proteome (Hubbard and Cohen, 1993; Graves and Haystead, 2003). In the heart, protein kinase activity is closely intertwined with different signal transduction cascades that regulate heart function in both pathologic and physiologic situations (Dhalla and Müller, 2010). By linking upstream signals with downstream regulatory pathways, protein kinases can affect a broad range of cellular functions including gene expression, membrane excitability and ion channels, cell cycle activity, and even metabolism (Bayer and Schulman, 2001; Rybakowska et al., 2015).

**FIGURE 1:**
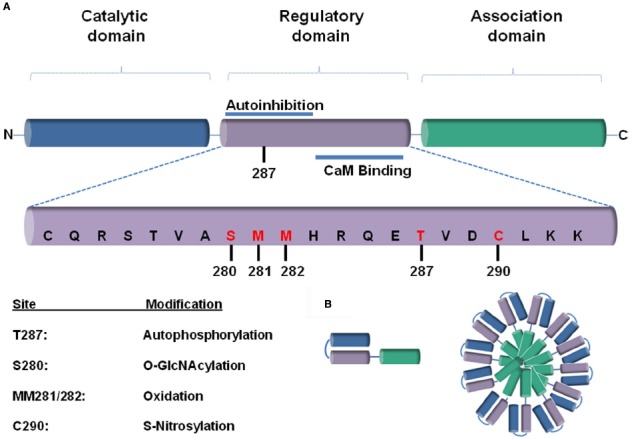
**(A)** A schematic representation of a CaMKII monomer, consisting of a (1) N-terminal catalytic domain, (2) regulatory domain, and (3) a C-terminal association domain. The most rigorously studied sites for different post-translational modifications (PTMs) are highlighted in red below. **(B)** The association domain (green), which is responsible for assembly into the holoenzyme, contains a hypervariable domain. Twelve to fourteen of these subunits multimerize together to form the CaMKII holoenzyme.

One of the well-investigated protein kinases that play a role in adaptive but presumably mainly in maladaptive remodeling processes in the heart is Ca^2+^-calmodulin-dependent protein kinase II (CaM Kinase II or CaMKII). CaMKII is a serine-threonine kinase with a putative *dodecameric* structure (Rosenberg et al., 2005) that consists of two *hexameric* stacked rings as previously described (Hudmon and Schulman, 2002; Anderson et al., 2011). Each CaMKII monomers consists of three subunits: a catalytic, a regulatory and an association one (Wang, 2008) that is responsible for the assembly into the holoenzyme (Rosenberg et al., 2006; Figure 1).

There are four different products of the CaMKII gene, with CaMKIIδ being the most abundantly expressed cardiac isoform (Hoch et al., 1998; Zhang et al., 2003; Anderson et al., 2011; Erickson et al., 2013; Gray and Heller Brown, 2014). CaMKII is critical for cellular processes such as excitation–contraction coupling (ECC), excitation–transcription coupling (ETC), chronotropic control via regulation of voltage-gated ion channels and Ca^2+^-handling proteins, sarcoplasmatic reticulum (SR) Ca ^2+^-uptake and release through ryanodine receptors (RyRs; Kushnir et al., 2010; Marx and Marks, 2013; Mattiazzi et al., 2015; Santulli and Marks, 2015).

CaMKII has been further shown to act as a molecular nexus linking neurohumoral stimulation to adverse cardiac remodeling and heart failure (HF) upon injury such as pressure overload, myocardial infarction and/or ischemia-reperfusion (IR; Maier and Bers, 2002; Zhang et al., 2003; Anderson et al., 2011; Erickson et al., 2013; Kreusser and Backs, 2014; Kreusser et al., 2014; Weinreuter et al., 2014; Capel and Terrar, 2015). Autonomous CaMKII activity seems to be increased in HF, and in patients with advanced and end-stage HF, there is a significantly up-regulated expression of CaMKII (Hoch et al., 1998; Maier, 2005). In heart disease, it may be also involved in cell death, inflammation (Weinreuter et al., 2014), disorders in ECC and ETC that may ultimately lead to cardiac dysfunction (Mattiazzi et al., 2015). Hyperactivity of CaMKII can also result in early after depolarizations (EADs) and proarrhythmic events (Zhang and Brown, 2004; Couchonnal and Anderson, 2008; Mishra et al., 2010; Schulman and Anderson, 2010).

As one example for phosphorylation targets, RyRs have been intensively studied. In the setup of HF, modifications of RyR2 lead to changes in the intracellular Ca^2+^ homeostasis that can further contribute to potentiation and/or perpetuation of maladaptive stress responses and proarrhythmic events. Whereas S2808 has been identified as a protein kinase A (PKA) site, CaMKII aims at S2814 (Ling et al., 2009; Marx and Marks, 2013; Heijman et al., 2014; Kreusser et al., 2014)—a modification which plays an important role in the regulation of the *force-frequency relationship* (FFR; Kushnir et al., 2010) in the heart. Murine models with RyR2-S2814A mutation were protected against pressure overload *in vivo* in the setting of transverse aortic constriction (TAC; Respress et al., 2012). In contrast, mice with a constitutive activation of the CaMKII site (S2814D) demonstrated exacerbated mortality (van Oort et al., 2010).

Interestingly, recent studies have argued that specific inhibition of CaMKII-mediated pathologic signaling might be an effective therapeutic strategy for addressing HF (Couchonnal and Anderson, 2008; Sossalla et al., 2010). Likewise, CaMKIIγ/δ knockout mice, in which CaMKII activity was completely abolished in the heart, were protected against cardiac dysfunction and interstitial fibrosis after pressure overload and β-adrenergic stimulation (Kreusser et al., 2014; Weinreuter et al., 2014).

Thus, the increasing mechanistic evidence underscores the need for development of highly specific pharmacological inhibitors for precise targeting of CaMKII maladaptive functions without affecting adaptive CaMKII-dependent signaling pathways, especially in the setting of HF.

### Post-Translational Modifications of Protein Kinases in Heart Disease

Myocardial homeostasis is dictated by the fragile balance between synthesis and degradation of proteins that control and mediate folding, trafficking to different compartments of the cell and the formation of protein complexes. Post-translational modifications (PTMs) are a highly effective mechanism for regulation of proteins by changing both structure and function of the affected targets. For enzymes, such as protein kinases, PTMs might either stimulate or suppress activity and thus dramatically affect downstream signaling.

Depending on mechanism of action, PTMs could be subdivided in three major groups (Liddy et al., 2013):

• Enzymatic: addition or subtraction of the modification is affected by proteins-such as phosphorylation, O- and N-linked glycosylation, acetylation, *S*-nitrosylation, sumoylation, etc. (Liddy et al., 2013).
• Chemical: e.g., alterations in the pH.
• Physical: e.g., cleavage or degradation of proteins, or interactions with other proteins, e.g., by building of signaling complexes.

In the following, we focus on some examples of PTMs that affect CaMKII function particularly in the heart.

### CaMKII and Phosphorylation

Phosphorylation of numerous protein kinases has been argued to be associated with the pathogenesis and progression of cardiovascular disease (Palaniyandi et al., 2009; Liu and Molkentin, 2011; Swaminathan et al., 2012) and has been shown to affect structure, function, as well as the intracellular compartmentalization of the target proteins. Extended Ca^2+^/CaM association with CaMKII leads to an increased autophosphorylation of CaMKII (e.g., CaMKIIδ at the T287 or CaMKIIα at the T286 site; Erickson, 2014). CaMKII autophosphorylation is important for its activation since it does not only enhance the affinity of CaM toward CaMKII (Meyer et al., 1992), but also prevents auto-inhibition of CaMKII even if Ca^2+^ decreases, thus conferring residual CaMKII activity after Ca/CaM dissociation (Lai et al., 1987).

Not only the specific activity of a kinase, but also its intracellular compartmentalization (cytosolic versus nuclear localization) depends on a variety of processes, including alternative splicing, or PTMs. The predominant CaMKII isoform in the heart—CaMKIIδ—exists in two splice variants—δB and δC—that differ in the presence or absence of a nuclear localization signal (NLS; Zhang and Brown, 2004; Mishra et al., 2011; Gray and Heller Brown, 2014). As a consequence, the δB isoform more likely affects gene expression whereas δC is thought to be more related to regulation of ECC (Zhang and Brown, 2004). However, neither splice variant is completely exclusive with respect to its nucleocytoplasmatic distribution as they exist in the same dodecamers with the consequence that the relative abundance in the complex may favor nuclear or cytosolic localization (Mishra et al., 2011). Moreover, as discussed below, phosphorylation of distinct spice variants may affect subcellular localization (Heist et al., 1998; Backs et al., 2006). In addition to this, we have shown that cytosolic CaMKII regulates gene expression by blocking nuclear import of histone deacetylase 4 (HDAC4; Backs et al., 2006; Zhang et al., 2007).

Nuclear targeting of CaMKII is regulated by (auto)phosphorylation (Heist et al., 1998; Bayer and Schulman, 2001; Backs et al., 2006; Rusciano et al., 2012) and might affect both downstream signaling and transcriptional regulation of CaMKII (Bayer and Schulman, 2001; Erickson et al., 2008).

## CaMKII and Oxidation

Heart failure and many of the conditions that predispose to it are associated with significant oxidative stress, which could both be maladaptive and leading to damage to membranes, proteins and DNA, but also adaptive by conveying specific regulatory effects (in terms of so-called “redox signaling”), or even activating physiological signaling pathways (Hafstad et al., 2013). Reactive oxygen species (ROS) have been reported to lead to a dynamic PTM at methionine residues (Erickson et al., 2008). *In vitro* studies in wild-type (WT) or mutant variants of Camui—the open form of CaMKII (Erickson et al., 2011)—reveal that angiotensin II (AngII) and endothelin-1 (ET1) activate CaMKII by a primarily oxidation-dependent pathway. Oxidation of M281/282 of CaMKII itself was shown to activate the kinase, thus underscoring the potential of oxidative stress to affect both pathologic and physiologic pathways in excitable cells, such as the cardiomyocyte. Upon Ca^2+^/CaM binding at the regulatory CaMKII domain, oxidation of M281/282 leads to activation of the enzyme (Erickson et al., 2008). Methionine oxidation inhibits re-association between regulatory and catalytic subunits of CaMKII (Chao et al., 2010) and thus enables perpetuation of CaMKII activity. Similarly, it has been demonstrated that sustained AngII stimulation results in activation of CaMKII, also increased cardiomyocyte p38 MAPK activation and apoptosis during transition to HF (Palomeque et al., 2009). Elevated levels of aldosterone in the circulation could also lead to an increased CaMKII oxidation, accompanied by pathological remodeling and cardiac dysfunction (He et al., 2011; Velez Rueda et al., 2012). In contrast, AngII–induced apoptosis was inhibited in isolated cardiomyocytes expressing oxidant-resistant CaMKII-mutant (Erickson et al., 2008).

Oxidation of CaMKII further affects sodium (Na^+^) transients (Wagner et al., 2011) and is thus associated with an increased susceptibility to arrhythmias due to altered electrical conductivity (Christensen et al., 2009; Swaminathan et al., 2011). A recent report showed that oxidative stress and mitochondrial ROS formation under the conditions of HF could also lead to oxidation and activation of CaMKIIδ and dysregulation of Ca^2+^ homeostasis independent from the Na^+^- induced Ca^2+^ overload (Viatchenko-Karpinski et al., 2014).

Recently, a CaMKII mutant which was resistant to oxidation modifications (MM281/282VV) was shown also to be protected upon myocardial infarction when compared to wildtype in the setup of diabetes (Luo et al., 2013). A MsrA (methionine sulfoxide reductase) knockout with higher levels of ox-CaMKII was showing dramatically reduced survival 30 days after myocardial infarction (Erickson et al., 2008) whereas MsrA overexpression seemed to be cardioprotective (He et al., 2011; Purohit et al., 2013). In addition to this, it has also been shown that activation of the CaMKII is causatively associated with contractile dysfunction in the setup of ER stress *in vivo* and that cardio-specific overexpression of catalase was sufficient to improve and attenuate ER stress-induced cardiac dysfunction (Roe and Ren, 2013).

## CaMKII and Nitrosylation

*S*-Nitrosylation is a common PTM and consists of *S*-nitrosothiol (SNO) formation from nitric oxide (NO; Choi et al., 2011). During *S*-nitrosylation, NO is further processed to dinitrogen trioxide (N_2_O_3_), accompanied by formation of SNO in the protein’s cysteine thiol residue (Gaston et al., 2003; Choi et al., 2011). A NO-“equivalent” is then exchanged between two interacting molecules (Lander et al., 1997; Stamler et al., 2001; Gaston et al., 2003; Choi et al., 2011).

*S*-Nitrosylation might lead to both activation and suppression of the activity of the affected proteins and so far target proteins have been identified as ion channels, transmembranous proteins and different transcriptional factors (Lander et al., 1997; Stamler et al., 2001; Lima et al., 2010; Choi et al., 2011). Recently, it has been shown that upon β-AR stimulation, CaMKII can be activated by increased production of nitric oxide (Gutierrez et al., 2013). *In vitro* application of 500 *μ*M L-N^G^-Nitroarginine methyl ester (L-NAME; an inhibitor of NO synthesis) in cardiomyocytes reduced Ca^2+^ sparks during diastole, whereas addition of the NO donor GSNO increased the rate of Ca^2+^ sparks and thus contributed to arrhythmias, possibly via sarcoplasmatic Ca^2+^ depletion (Gutierrez et al., 2013). C290 has been pointed out as a predicted CaMKII *S*-nitrosylation site, although the exact mechanism by which nitrosylation induces CaMKII activity in the cardiomyocyte is still unclear (Gutierrez et al., 2013).

## CaMKII and *O*-GlcNAcylation

Diabetes mellitus is also a disease where CaMKII activation has been observed: for example, there is strong evidence indicating that an increased ox-CaMKII to CaMKII ratio upon myocardial infarction is associated with higher mortality in diabetic patients (Luo et al., 2013) in comparison to non-diabetic ones.

Recently, it has been shown that not only oxidation, but also O-linked glycosylation (*O*-GlcNAcylation) via activation of the hexosamine biosynthetic pathway (HBP) might enhance CaMKII activity. *O*-GlcNAcylation at S279 might in its turn lead to an autonomous activation of CaMKII, creating a molecular memory even after Ca^2+^-concentration in the cell has declined (Erickson et al., 2013). High glucose levels induce CaMKII activation as well as spontaneous SR Ca^2+^ sparks that can lead to arrhythmias and contraction impairment in NRVMs (Erickson et al., 2013). Blocking of *O*-GlcNAcylation via DON-a pharmacologic HBP inhibitor—was sufficient to decrease abnormal CaMKII activity and heart beat irregularities in diabetic animals (Erickson et al., 2013). Further studies are warranted to learn more about the relative importance of *O*-GlcNAcylation versus oxidation of CaMKII in metabolic disease. It will be also of particular interest whether distinct modifications lead to activation of CaMKII in different cellular compartments and could thus modulate the affinities to certain targets.

## Cross-Talk Between Different PTMs

In recent years, there is an overwhelming appreciation for the diversity of PTMs, but the complex interplay between them is incompletely understood. In one recent study (Erickson et al., 2013), *O*-GlcNAc modification of CaMKII was also linked to enhanced CaMKII phosphorylation in cardiomyocytes during periods of elevated glucose levels. Although there is an increasing evidence for an extensive cross-talk taking place between different PTMs on example of CaMKII, more elaborated work such as computational modeling is needed to understand the relative contribution of these interactions in the context of cardiac health and disease.

Table 1 indicates the most often encountered PTM-sites within the regulatory unit of CaMKII and highlights the regulatory pathways and disease states that affect those modifications.

**TABLE 1:** **The most important PTMs affecting CaMKII function in the heart in terms of the modification sites, the conditions, under which PTMs are observed and their effect in the heart**.

**Modification**	**Site**	**Disease/stimuli**	**Effect**	**Citation(s)**
Phosphorylation	T286/287 S332	Atrial fibrillation, sympathetic stimulation	Perpetuated activation Blocked nuclear signaling	Lai et al. (1987), Heist et al. (1998), Bayer and Schulman (2001)
Oxidation	M280/281	ROS, diabetes,	Activation angiotensin II injury	Palomeque et al. (2009), Swaminathan et al. (2011), Luo et al. (2013), Purohit et al. (2013)
*O*-GlcNAcylation	S279/280	Diabetes	Activation	Erickson et al. (2013)
*S*-Nitrosylation	C290	β-Adrenergic receptor (β-AR) stimulation	Activation, ryanodine receptor phosphorylation	Choi et al. (2011)

## Interaction of Other Pathways With CaMKII Signaling

The close interaction between PKA and CaMKII is known for a long time (Haghighi et al., 2014). Recently, it has been reported that CaMKII affects cAMP levels both under normal conditions and upon β-adrenergic stimulation (Mika et al., 2015) via regulation of PDE4D (phosphodiesterase 4D).

Another example for the extensive cross-talk between PKA and CaMKII pathways has recently revealed that both signaling pathways are not mutually exclusive, but often complementary to each other (Pereira et al., 2013). In this paper, the authors claim that Epac (exchange protein activated by cAMP) activation upon β-adrenergic stress can lead to arrhythmogenic SR Ca^2+^ release that involves increased phosphorylation of the RyR by CaMKII (Pereira et al., 2013). In particular, the Epac2 isoform was specifically shown to mediate Ca^2+^ release from the SR in a PKA-independent fashion by S2814 phosphorylation (Pereira et al., 2013).

The extensive interactions between PKA and CaMKII signaling pathways with far-reaching consequences on the cardiac genome could also be demonstrated on their effects on different members of the HDAC family, such as HDAC5. Acute β-adrenergic stimulation and/or PKA activation has been shown to enable nuclear retention of HDAC5 (Ha et al., 2010; Sucharov et al., 2011; Haworth et al., 2012; Chang et al., 2013). During acute adrenergic stress, PKA activation could at least party dominate over CaMKII/PKD signaling pathways to provide a short time frame for protection against damage. However, there is still a controversy as to whether PKA leads to HDAC5 nuclear retention by direct phosphorylation of HDAC5 (Ha et al., 2010; Chang et al., 2013) or whether PKA leads to HDAC5 nuclear retention via phosphorylation-independent (Haworth et al., 2012) or indirect (Sucharov et al., 2011) mechanisms, e.g., via inhibition of PKD. Future work is needed to clarify these different findings/hypotheses.

Moreover, our recent data unmasked a PKA-dependent signaling pathway by which PKA overcomes CaMKII-dependent epigenetic signaling toward HDAC4 (Backs et al., 2011). PKA induces proteolysis of HDAC4, generating a stably expressed N-terminal HDAC4 fragment (HDAC4-NT) that could in turn selectively enter the nucleus and suppress gene programs which drive pathological remodeling (such as the transcriptional factor MEF2), without affecting cardiomyocyte survival. Remarkably, HDAC4-NT is resistant to CaMKII because it lacks the CaMKII phosphorylation sites: a finding which might facilitate the development of NT-based therapeutic approaches for circumventing pathological CaMKII signaling.

On the other hand, activated CaMKII stably associates with full-length HDAC4, but not directly with HDAC5, which responds only to CaMKII when associated with HDAC4 (Backs et al., 2006, 2008). This implies that the interaction between CaMKII and HDAC4 is critical for downstream transcriptional regulation in response to Ca^2+^ signaling; for example, a CaMKII-non-responsive HDAC4 mutant was resistant to prohypertrophic signaling (Backs et al., 2006). Further screening for identifying pharmacological inhibitors that interrupt specifically this CaMKII/HDAC4 interaction is underway Backs et al. (unpublished).

Another group of proteins that closely interact with CaMKII are beta-arrestins (Reiter et al., 2012). Upon binding to G-protein-coupled receptors (GPCRs), they function as adaptor molecules that can scaffold numerous proteins involved in cardiovascular diseases, e.g., cAMP phosphodiesterase, PDE4D, kinases such as ERK1/2, and CaMKIIδ (Perry et al., 2002; Wu et al., 2006; Xiao et al., 2007; Gurevich et al., 2008). *In vivo* models of HF suggest a beneficial function of β-arrestin-mediated ERK stimulation via both β-1-adrenergic and AngII Type 1A receptors (Tohgo et al., 2003; Zhai et al., 2005; Noma et al., 2007; Gurevich et al., 2008; Chiara Andreoli, 2013). In contrast, β-arrestin-mediated activation of CaMKIIδ can trigger maladaptive events (Mangmool et al., 2010; Bathgate-Siryk et al., 2014). Due to the selective effects of β-arrestin on cardiac homeostasis, future therapeutic efforts might focus on development of the so-called “biased ligands” that could counteract maladaptive and stimulate adaptive G-protein signaling (Patel et al., 2008).

Furthermore, recent observations on the role of CaMKII in left ventricular hypertrophy in the setup of hypertension have provided some interesting evidence regarding the reciprocal activation of CaMKII and ERK (extracellular regulated kinase; Cipolletta et al., 2015). In this particular study, *in vivo* treatment with inhibitors of CaMKII lead to an inhibition of cardiomyocyte hypertrophy-a finding which went along with a reduction of CaMKII and ERK phosphorylation and nuclear accumulation (Cipolletta et al., 2015).

### miRNAs

It has been also recently reported that CaMKII could be regulated via different microRNAs, such as miR-145 and that miR-145 affects CaMKIIδ-regulated transcriptional programs (Cha et al., 2013). Another member of the micro RNA family: miR-214 has been shown to be cardioprotective in the setup of IR injury by counteracting Ca^2+^ overload in response to impairment of coronary blood flow through inhibition of CaMKIIδ—the main CaMKII isoform in the heart (Aurora et al., 2012).

### Other Modifications

Whereas the role of further modifications—such as ubiquitination of CaMKII have been implicated to mediate in the synaptic plasticity in neurons (Tsai, 2014), similar modifications and the role of the proteasome in the regulation of CaMKII activity in the heart still remain to be further elucidated.

### Proteolysis

Calpains catalyze the proteolysis of numerous and diverse cytosolic, cytoskeletal, and membrane-associated substrates in response to cell injury *in vivo* and *in vitro* (Tremper-Wells and Vallano, 2005). In primary neuronal cultures, calpain-mediated proteolysis of nuclear CaMKIV under conditions of sustained Ca^2+^ activity affects CaMK-dependent gene transcription, thereby preventing excessive transcriptional response (Tremper-Wells and Vallano, 2005). Proteolysis has not been directly investigated as a possible mediator of CaMKII activity in the heart yet; however, the increasing evidence from investigations in the neurovascular field suggests that there are still plenty of unexplored mechanisms of CaMKII regulation that extend beyond our current scope of knowledge. Although CaMKIV has been long thought to be a kinase with expression pattern limited only to neuronal tissue, recently it was shown that this particular CaMK—variant contributes to controlling nitric oxide synthase (NOS) activity in endothelial cells (Santulli et al., 2012)—a finding that uncovered a previously unknown mechanism for CaMK-dependent blood pressure control. A genome-wide analysis done several years ago within the Framingham Heart Study 100K Project (Larson et al., 2007; Santulli et al., 2012) has pointed out an important links between vessel wall stiffness and variants of the human CaMKIV gene and thus opened room for discussions regarding the role of other CaMK family members in the regulation of cardiovascular health and disease.

## Summary

The mammalian heart responds to perpetuated stress with a remodeling process that is characterized by myocyte hypertrophy, alterations in ion currents and impaired contractility that ultimately culminate in HF. Among the plethora of molecular changes that occur during pathological cardiac remodeling alterations in the expression, structure, enzymatic activity and/or sub-cellular localization of a number of protein kinases are often critically involved in maladaptive signaling.

As a member of the protein kinase family, CaMKII represents an important nodular molecule translating different types of stress into both pathological and physiological downstream pathways in the heart and has therefore emerged as a promising therapeutic target in the context of HF. A profound understanding of CaMKII’s regulatory mechanisms under different stress situations might enable the future development of pathway-or disease-specific CaMKII-based therapies that combine high treatment efficacy with low potential for off-target effects.

### Conflict of Interest Statement

The authors declare that the research was conducted in the absence of any commercial or financial relationships that could be construed as a potential conflict of interest.

## Abbreviations

β-AR: beta-adrenergic receptor
CaMKII: calcium/calmodulin-dependent protein kinase II
DON: 6-diazo-5-oxo-1-norleucine
EAD: early after depolarization
ECC: excitation–contraction coupling
Epac: exchange protein activated by cAMP
ETC: excitation–transcription coupling
FFR: force-frequency relationship
GPCR: G-protein-coupled receptor
HBP: hexosamine biosynthetic pathway
HDAC: histone deacetylase
HF: heart failure
IR: ischemia-reperfusion
L-NAME: L-N^G^-Nitroarginine methyl ester
MsrA: methionine sulfoxide reductase A
NOS: nitric oxide synthase
PDE4D: phosphodiesterase 4D
PKA: protein kinase A—cyclic AMP-dependent protein kinase
PTM: post-translational modification
RyR: ryanodine receptor
SNO: *S*-nitrosothiol.
